# EasyLB: Adaptive Load Balancing Based on Flowlet Switching for Wireless Sensor Networks

**DOI:** 10.3390/s18093060

**Published:** 2018-09-12

**Authors:** Zhiqiang Guo, Xiaodong Dong, Sheng Chen, Xiaobo Zhou, Keqiu Li

**Affiliations:** Tianjin Key Laboratory of Advanced Networking, School of Computer Science and Technology, Tianjin University, Tianjin 300350, China; guozhiqiang@tju.edu.cn (Z.G.); dongxiaodong@tju.edu.cn (X.D.); chensheng@tju.edu.cn (S.C.); likeqiu@gmail.com (K.L.)

**Keywords:** wireless sensor networks, flowlet switching, load balancing, Markov chain, software defined networking

## Abstract

Load balancing is effective in reducing network congestion and improving network throughput in wireless sensor networks (WSNs). Due to the fluctuation of wireless channels, traditional schemes achieving load balancing in WSNs need to maintain global or local congestion information, which turn out to be complicated to implement. In this paper, we design a flowlet switching based load balancing scheme, called EasyLB, by extending OpenFlow protocol. Flowlet switching is efficient to achieve adaptive load balancing in WSNs. Nevertheless, one tricky problem lies in determining the flowlet timeout value, δ. Setting it too small would risk reordering issue, while setting it too large would reduce flowlet opportunities. By formulating the timeout setting problem with a stationary distribution of Markov chain, we give a theoretical reference for setting an appropriate timeout value in flowlet switching based load balancing scheme. Moreover, non-equal probability path selection and multiple parallel load balancing paths are considered in timeout setting problem. Experimental results show that, by setting timeout value following the preceding theoretical reference, EasyLB is adaptive to wireless channel condition change and achieves fast convergence of load balancing after link failures.

## 1. Introduction

In wireless sensor networks (WSNs), many sensor nodes are deployed to collect various types of data from the environment, e.g., temperature, image and video. The data collected are forwarded to the sink nodes through wireless channels with or without the help of some intermediate nodes. At the sink nodes, the data are further processed to perform some specific tasks, such as fire detection, water quality monitoring and natural disaster prevention. To improve reliability and throughput, multi-path routing algorithms are widely used in WSNs [1,2,3,4,5,6], where load balancing plays a key role in reducing transmission latency and extending the lifetime of WSNs. As the wireless channels fluctuate frequently due to the inherent characteristics of wireless signals, the channel capacities of the paths change rapidly [7,8]. In the worst case, some links may even fail if deep fade happens [9]. In this scenario, how to achieve adaptive load balancing in WSNs is a crucial problem.

Many efforts focus on achieving load balancing in various scenarios. These load balancing schemes differ in operation granularity and the ability to handle asymmetry. According to load balancing granularity, load balancing schemes can be broadly divided into three categories: packet level, flow level and sub-flow level. MPTCP [10], DRB [11], Fastpass [12] and DeTail [13] are typically working at packet level. In general, the packet level schemes are able to achieve accurate control of load ratio among multi-paths, but often lead to packet reordering. ECMP [14], WCMP [15], Hedera  [16] and MicroTE [17] operate load balancing at flow level. These schemes do not generally cause packet reordering problem, but they cannot achieve accurate load balancing ratio. Flare [18], Presto [19], Conga [20] and LetFlow [21] are sub-flow level load balancing schemes that obtain a trade-off between accurate ratio control and freedom from packet reordering.

Load balancing in symmetrical network topologies such as Fat-tree [22] and Clos [23] usually splits network traffic across multiple equal paths which have the same link capacity and delay. Due to the fluctuation of wireless channels, asymmetries may occur frequently in WSNs where the capacities of parallel routing paths are no longer the same. Many existing load balancing schemes can work normally in symmetric networks. However, they will result in serious network congestion and a sharp network performance decline in WSNs when asymmetries occur, such as ECMP. Load balancing schemes can work in an asymmetric network when network congestion information is available, such as CONGA [20] and HULA [24]. However, the implementation of CONGA or HULA in WSNs is very complex because they need to obtain real-time congestion information with a centralized control of fabric and end-to-end feedback. Recently, Vanini, et al. [21] verified that adaptive load balancing can be implemented in asymmetric topology based on flowlet switching [25]. This scheme is extremely simple to implement without any explicit congestion feedback.

**Figure 1:**
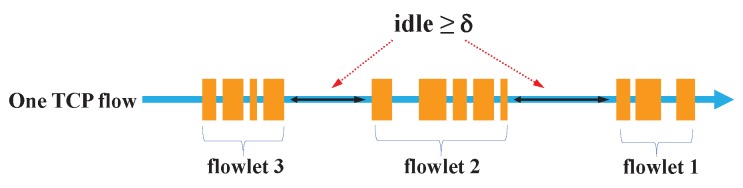
Flowlet is a burst of packets that is separated in time from other bursts in one TCP flow by an idle time interval that is larger than a predefined timeout value, δ.

**Figure 2:**
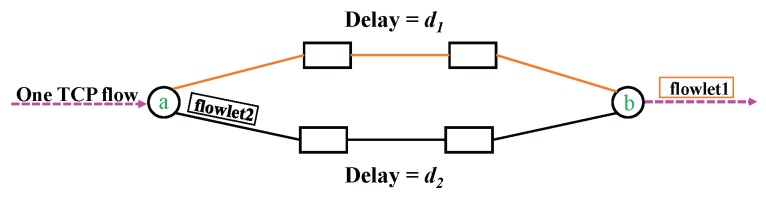
Flowlet switching: the packets in one flowlet follow the same path. If $\delta \geq \left|d_{1}-d_{2}\right|$, one can randomly assign the packets in flowlet2 to one path without risking TCP packet reordering. Since packets in flowlet1 will always leave the merge point b before flowlet2.

As shown in Figure 1, a flowlet is a sub-flow which consists of several consecutive packets from the same TCP flow. Flowlets are characterized by a timeout value, δ, which is the minimum inter-flowlet interval, i.e., packet interval within a flowlet is smaller than δ. Flowlets can be switched independently. Flowlet switching based load balancing will not cause TCP reordering if δ is set greater than the maximum delay difference between any set of parallel paths, as shown in Figure 2.

The size of flowlet will change with the real-time network congestion. More specifically, the less congested the network is, the larger the size of flowlet is. Thus, Vanini et al. [21] found that this property of flowlet can achieve adaptive and resilient load balancing in the presence of topological asymmetry. They have implemented an extremely simple flowlet switching based load balancing scheme, called LetFlow [21]. However, a key problem lies in determining a proper timeout value. Setting the value too small will lead to heavy packet reordering issue, and setting the value too large will reduce the opportunities of flowlet generation.

However, to achieve adaptive load balancing, choosing a proper timeout value is quite difficult because of dynamic TCP traffic patterns, different capacities of multiple parallel paths, etc. Currently, the timeout is usually set empirically based on sufficient simulations and statistics. Besides, a pre-designed timeout in one network cannot always reach good performance in other scenarios. To solve this problem, in this paper, we investigate the theoretical reference on setting an appropriate timeout value in flowlet switching based load balancing scheme, and verify the theoretical results in an OpenFlow enabled network. The contributions of this paper are summarized as follows:

- First, we design a flowlet switching based load balancing scheme for WSNs, called EasyLB, by adding one selection method for group table defined in OpenFlow [26]. We show that, by setting timeout according to the theorem, EasyLB achieves approximately optimal load balancing in WSNs and re-balances traffic quickly after link failures.
- Second, the flowlet switching process is modeled by a stationary Markov chain, with the assumption that all flows occur as the Poisson process. Based on this model, we derive a theorem that specifies the sufficient condition on timeout that ensures the system can converge to an ideal load balancing effect.
- Third, we further study the timeout setting problem when it comes to non-equal probability path selection and multiple parallel paths in flowlet switching. We conclude more generally when non-equal probability path selection is adopted in flowlet switching based load balancing scheme. Then, we give an algorithm for solving timeout setting problem in multiple parallel load balancing paths.

The paper is organized as follows. Section 2 presents the design and implementation of EasyLB. In Section 3, we describe the flow number change of parallel paths with a Markov chain model, and then we reveal the relationship between δ and the load balancing effect by the stationary distribution of Markov chain. We further study the timeout setting problem from the perspective of non-equal probability path selection and multiple parallel paths in flowlet switching. We evaluate EasyLB in different scenarios in Section 4. Section 5 concludes the paper and presents our future works.

## 2. EasyLB Design and Implementation

In this section, we introduce the architecture of EasyLB and give a brief explanation of the modified group table selection algorithm. EasyLB can be deployed at any merge nodes in WSNs.

**Figure 3:**
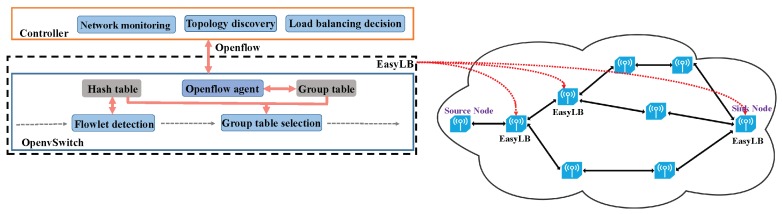
An overview of EasyLB and its deployment policy in wireless sensor networks.

The architecture of EasyLB and one example of deployment policy in WSNs is shown in Figure 3. In the WSN, the sensor nodes are reconfigurable, which is enabled by software defined networking. More specifically, the sensor nodes communicate with a common controller via OpenFlow [26]. Flowlet detection module of EasyLB is implemented in the sensor nodes while load balancing decision module resides in the controller. The controller obtains the whole network topology through the topology discovery module and periodically collects the channel state, link delay and flow information through the network monitoring module. The controller pushes the source-destination multi-path decisions into corresponding sensor nodes as group table entries.

As one representative southbound protocols in software defined networking [27], OpenFlow [26] standardizes the control signalling between control plane and data plane. Group table defined in OpenFlow protocol consists of multiple group entries and provides more advanced packet forwarding features to OpenFlow enabled switches. Each group entry contains multiple action buckets. Only one action bucket chosen by selection method such as hash will be executed in the select type of group entry. Obviously, the group entry of type “select” is suitable for implementing multi-path forwarding. By extending the selection method, we implement EasyLB by modifying the source code of OpenvSwitch  [28].

The group table selection algorithm is described in Algorithm 1; the hash tables of *last_arrival_time* and *last_output_channel* are used to record the last packet arrival time and egress channel for every flow, respectively. When the time interval between two packets belonging to one flow is greater than δ, one new flowlet is created. The flowlet switching will randomly choose one from available channels of multi-path as its egress channel. If time interval is smaller than δ, the packet will still be forwarded from the same egress channel as the previous one of the same flow. The *group_alive_buckets* function selects a normal state channel from available output channels. When channel is down, the corresponding action bucket will not be selected or executed. Meanwhile, a port down message will be sent to controller and controller will make a new multi-path decision. These mechanisms can guarantee the fast convergence of load balancing after link failures.

**Algorithm 1** The group table selection algorithm.
1:**procedure TABLESELECT**2:last_arrival_time ← None;3:last_output_channel ← None;4:timeout ←δ;5:**while** receive one packet **do**6: *key ← hash(packet);*7: *current_time ← getTimeNow();*8: **if** *key* not in keys of *last_arrival_time* **then**9:  add (key,current_time) to last_arrival_time;10:  output_channel ← random(group_alive_buckets());11:  add (key,output_channel) to last_output_channel;12: **else**13:  last_time ← get(key,last_arrival_time);14:  **if** current_time-last_time≥timeout **then**15:    update (key,current_time) to last_arrival_time;16:    output_channel←random(group_alive_buckets());17:    update (key,output_channel) to last_output_channel;18:  **else**19:    update(key,current_time) to last_arrival_time;20:    last_channel←get(key,last_output_channel);21:    **if** last_channel corresponding bucket is not alive **then**22:      output_channel←random(group_alive_buckets());23:      update (key,output_channel) to last_output_channel;24:      send one port down message to controller;25:    **else**26:      output_channel←last_channel;27:    **end if**28:  **end if**29: **end if**30: send packet to output_channel;31:**end while**32:**end procedure**


## 3. Timeout Setting in EasyLB

A wrongly chosen timeout value cannot mitigate the congestion or even reduce the throughput of the whole network in flowlet switching based load balancing scheme. Generally, the value of timeout is obtained in advance through sufficient simulations and statistics. In this section, we introduce the Markov chain model to describe the flowlet switching process, and to investigate the timeout setting problem in EasyLB. We first start with the simplest case where there are two parallel paths with equal path selection, and then extend it to non-equal path selection scenario. Finally, we solve the timeout setting problem in the general multiple parallel case.

### 3.1. Markov Chain Model

Without loss of generality, consider that *N* flows transfer on two parallel paths *P*${}_{1}$ and *P*${}_{2}$ with bottleneck capacities *C*${}_{1}$ and *C*${}_{2}$, respectively. Assume the arrivals of packets of each flow follow the Poisson process (although not all flows are subject to Poisson process in different network environments, the assumption of Poisson process can help us better understand the burstiness of TCP and carry out relevant theoretical studies, as shown in [29,30,31,32]), and all the competing flows on the same path fairly share the path’s capacity. Let $F_{1}$ and $F_{2}$ denote the set of flows on *P*${}_{1}$ and *P*${}_{2}$, respectively. In an instant, we use γ to represent the average packet size, thus the arrival rate $\lambda_{i}$ of flow *i* can be calculated as

$$\lambda_{i}=\left\{\begin{array}{cc} \frac{C_{1}}{n_{1}\gamma }, & i\in F_{1},\\ \frac{C_{2}}{n_{2}\gamma }, & i\in F_{2}, \end{array}\right.$$

where $n_{1}=\;|F_{1}|$ and $n_{2}=\;|F_{2}|$ denote the number of flows on $P_{1}$ and $P_{2}$, respectively. The aggregate arrival rate $\lambda_{a}$ on *P*${}_{1}$ and *P*${}_{2}$ is

$$\lambda_{a}=\frac{C_{1}+C_{2}}{\gamma }.$$

**Figure 4:**
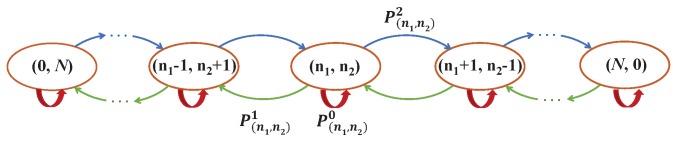
The model of Markov chain. In state ($n_{1},n_{2}$), there are $n_{1}$ and $n_{2}$ flows on $P_{1}$ and $P_{2}$, respectively. The green line represents flow *i* on $P_{1}$ triggers a new flowlet and selects $P_{2}$ as its next path while the blue line represents flow *i* on $P_{2}$ triggers a new flowlet and selects $P_{1}$ as its next path. The red line represents the other cases.

Obviously, the flowlet switching process can be modeled by a Markov Chain, where the number of flows on $P_{1}$ and $P_{2}$ is the state. For state ($n_{1}$, $n_{2}$), the next state it may transfer into is ($n_{1}$, $n_{2}$), ($n_{1}$ − 1, $n_{2}$ + 1) or ($n_{1}$ + 1, $n_{2}$ − 1), which depends on the random path selection of new triggered flowlet. As Figure 4 depicts, if flow *i* on path $P_{1}$ triggers a new flowlet and the random selection path is $P_{2}$, ($n_{1}$, $n_{2}$) will transfer to ($n_{1}$ − 1, $n_{2}$ + 1). Similarly, if flow *i* on path $P_{2}$ triggers a new flowlet and the random selection path is $P_{1}$, ($n_{1}$, $n_{2}$) will transfer to ($n_{1}$ + 1, $n_{2}$ − 1). Otherwise, the state will remain unchanged.

Let $P_{\left(n_{1},n_{2}\right)}^{0}$, $P_{\left(n_{1},n_{2}\right)}^{1}$ and $P_{\left(n_{1},n_{2}\right)}^{2}$ denote the transition probability from state ($n_{1}$, $n_{2}$) to ($n_{1}$, $n_{2}$), ($n_{1}$ − 1, $n_{2}$ + 1) and ($n_{1}$ + 1, $n_{2}$ − 1), respectively. According to [21], the transition probability $P_{\left(n_{1},n_{2}\right)}^{1}$ and $P_{\left(n_{1},n_{2}\right)}^{2}$ can be calculated as

$$P_{\left(n_{1},n_{2}\right)}^{1}=\frac{1}{2}\sum_{i\in F_{1}}\left(f\left(\lambda_{i}\right)+g\left(\lambda_{i}\right)\right),\;n_{1}\in \left[1,N\right],$$

and

$$P_{\left(n_{1},n_{2}\right)}^{2}=\frac{1}{2}\sum_{i\in F_{2}}\left(f\left(\lambda_{i}\right)+g\left(\lambda_{i}\right)\right),\;n_{1}\in \left[0,N-1\right],$$

where $f\left(\lambda_{i}\right)=\frac{\lambda_{i}}{\lambda_{a}-\lambda_{i}}\left(e^{-\lambda_{i}\delta }-e^{-\lambda_{a}\delta }\right)$ and $g\left(\lambda_{i}\right)=\frac{\lambda_{i}}{\lambda_{a}}e^{-\lambda_{a}\delta }$.

Since $\lambda_{i}\ll \lambda_{a}$, $P_{\left(n_{1},n_{2}\right)}^{0}$, $P_{\left(n_{1},n_{2}\right)}^{1}$ and $P_{\left(n_{1},n_{2}\right)}^{2}$ can be approximated as

$$\begin{array}{cc} P_{\left(n_{1},n_{2}\right)}^{1}\approx & \frac{C_{1}}{2(C_{1}+C_{2})}e^{-\frac{C_{1}\delta }{n_{1}\gamma }},\;n_{1}\in \left[1,N\right],\\ P_{\left(n_{1},n_{2}\right)}^{2}\approx & \frac{C_{2}}{2(C_{1}+C_{2})}e^{-\frac{C_{2}\delta }{n_{2}\gamma }},\;n_{1}\in \left[0,N-1\right],\\ P_{\left(n_{1},n_{2}\right)}^{0}= & 1-P_{\left(n_{1},n_{2}\right)}^{1}-P_{\left(n_{1},n_{2}\right)}^{2},\;n_{1}\in \left[1,N-1\right]. \end{array}$$

Note that the transition probabilities of $P_{\left(0,N\right)}^{0}$ and $P_{\left(N,0\right)}^{0}$ are $1-P_{\left(0,N\right)}^{2}$ and $1-P_{\left(N,0\right)}^{1}$, respectively.

The transition probability between ($n_{1}$, $n_{2}$) and any other state besides ($n_{1}$, $n_{2}$), ($n_{1}$ − 1, $n_{2}$ + 1) and ($n_{1}$ + 1, $n_{2}$ − 1) is 0. In summary, the transition probability matrix P of this Markov chain can be written  as

$$P={\left[\begin{array}{c} \begin{array}{ccccc} P_{\left(0,N\right)}^{0} & P_{\left(0,N\right)}^{2} & & & \\ P_{\left(1,N-1\right)}^{1} & P_{\left(1,N-1\right)}^{0} & P_{\left(1,N-1\right)}^{2} & & \\ & \ddots & \ddots & \ddots & \\ & & P_{\left(N-1,1\right)}^{1} & P_{\left(N-1,1\right)}^{0} & P_{\left(N-1,1\right)}^{2}\\ & & & P_{\left(N,0\right)}^{1} & P_{\left(N,0\right)}^{0} \end{array} \end{array}\right]}_{\;}$$

which is a typical tridiagonal matrix.

### 3.2. Formalization of Timeout Setting Problem

In this subsection, we derive the sufficient condition on timeout that ensures the flowlet switching based load balancing scheme to achieve ideal load balancing effect.

**Definition** **1.** In the flowlet switching based load balance scheme considered in the previous section, if $\frac{n_{1}}{n_{2}}=\frac{C_{1}}{C_{2}}$, the ideal load balancing effect is achieved.

We define μ as the number of flows mapping to $P_{1}$ when the ideal load balancing effect is achieved. According to Definition 1, μ = $\left\lfloor \frac{C_{1}}{C_{1}+C_{2}}\times N\right\rfloor$. In this case, the corresponding state of the Markov chain is (μ,N−μ), we call it the *ideal state*.

**Theorem** **1.** *In flowlet switching based load balancing scheme, if N flows transmit over two parallel paths with capacities $C_{1}$ and $C_{2}$, respectively, the sufficient condition on timeout δ to achieve ideal load balancing effect is δ > $\delta_{\min}$, where*

(1) $$\delta_{\min}=\left\{\begin{array}{cc} 0, & \;if\;C_{1}=C_{2},\\ \frac{\mu \left(N-\mu +1\right)\gamma \times \ln\left(\frac{C_{1}}{C_{2}}\right)}{C_{1}\left(N-\mu +1\right)-C_{2}\mu }, & \;if\;C_{1}>C_{2},\\ \frac{\left(N-\mu \right)\left(\mu +1\right)\gamma \times \ln\left(\frac{C_{1}}{C_{2}}\right)}{C_{1}\left(N-\mu \right)-C_{2}\left(\mu +1\right)}, & \;if\;C_{1}<C_{2}. \end{array}\right.$$

**proof** **of** **Theorem** **1.** The number of states in the preceding Markov chain is restricted and all states are accessible, thus this Markov chain is irreducible and all states are recurrent. Additionally, any state can access itself again through one step, so this Markov chain has stationary distribution. Let $\vec{\pi }=\left[\pi_{0},\pi_{1},\ldots ,\pi_{m},\ldots ,\pi_{N}\right]$ represent the stationary distribution of this Markov chain, where $\pi_{m}$ denotes the stationary probability of state (m,N−m). We can get the stationary distribution by solving

$$\vec{\pi }P=\vec{\pi }\;$$

where $\sum_{m=0}^{N}\pi_{m}=1$. After some mathematical manipulations, we finally get:

(2) $$\begin{array}{c} \frac{\pi_{m}}{\pi_{m+1}}=\frac{P_{\left(m+1,N-m-1\right)}^{1}}{P_{\left(m,N-m\right)}^{2}}\;\;\;0\leq m\leq N-1. \end{array}$$

The relationship of stationary probability between *ideal state* and that of any other state can be deduced from Equation (2) as:

(3) $$\begin{array}{c} \frac{\pi_{\mu }}{\pi_{\mu +k}}=\frac{\pi_{\mu }}{\pi_{\mu +1}}\times \frac{\pi_{\mu +1}}{\pi_{\mu +2}}\times ....\times \frac{\pi_{\mu +k-1}}{\pi_{\mu +k}}\\ =(\frac{C_{1}}{C_{2}}{)}^{k}e^{-\delta \sum_{j=1}^{k}\left(\frac{C_{1}}{(\mu +j)\gamma }-\frac{C_{2}}{(N-\mu -j+1)\gamma }\right)},\;k\in \left[1,N-\mu \right], \end{array}$$

(4) $$\begin{array}{c} \frac{\pi_{\mu -k}}{\pi_{\mu }}=\frac{\pi_{\mu -1}}{\pi_{\mu }}\times \frac{\pi_{\mu -2}}{\pi_{\mu -1}}\times ....\times \frac{\pi_{\mu -k}}{\pi_{\mu -k+1}}\\ =(\frac{C_{1}}{C_{2}}{)}^{k}e^{-\delta \sum_{j=1}^{k}\left(\frac{C_{1}}{(\mu -j+1)\gamma }-\frac{C_{2}}{(N-\mu +j)\gamma }\right)},\;k\in \left[1,\mu \right]. \end{array}$$

From the perspective of stationary distribution, when it comes to ideal load balancing effect, the stationary probability of *ideal state* is greater than any other one, i.e.,

(5) $$\begin{array}{cc} \frac{\pi_{\mu }}{\pi_{\mu +k}} & >1,\;\forall \;k\in [1,N-\mu ], \end{array}$$

(6) $$\begin{array}{cc} \frac{\pi_{\mu -k}}{\pi_{\mu }} & <1,\;\forall \;k\in [1,\mu ]. \end{array}$$

By solving Equations (5) and (6), we can obtain the results specified in Equation (1). For more detailed derivation, please refer to Appendix A. ☐

When the capacity of $P_{1}$ is greater than that of $P_{2}$, Equation (3) is always larger than 1. That is, when $P_{1}$ has more flows than *ideal state*, flows themselves incline to transfer to $P_{2}$. Contrarily, when the capacity of $P_{1}$ is smaller than that of $P_{2}$, Equation (4) is always smaller than 1. This indicates that, when $P_{1}$ has fewer flows than *ideal state*, flows tend to transfer to $P_{1}$. Note that, when paths are symmetric, the timeout has no effect on the final load balancing state.

Assuming that the delay difference between $P_{1}$ and $P_{2}$ is *d*, δ not only has to meet the constraints in Theorem 1, but also has to be greater than *d* to avoid packet reordering. However, setting δ too large also lowers the possibility of flowlet generation. An extreme case is setting δ at infinity, which will result in a flow-level load balancing scheme. An upper bound on δ is left as a future study.

### 3.3. Timeout Setting in Non-Equal Probability Path Selection

It is shown in [21] that, with equal probability path selection, flowlet switching based load balancing scheme can achieve adaptive load balancing on the premise of appropriate selection of δ, which is the magic of this load balancing technology. Considering a more general case where the paths are selected with non-equal probabilities, it is worth investigating whether the flowlet switching based load balancing scheme can still maintain its adaptive load balancing capability. In that case, how should δ be set? In practice, the paths are usually selected with non-equal probabilities; let us take the following two scenarios as an example. The first scenario is the switch queue’s length on path $P_{1}$ is greater than that on path $P_{2}$. To reduce the packet loss rate and improve overall performance of the network, we should choose path $P_{1}$ as new routing path at a higher probability for newly triggered flowlet. The second scenario is when the bandwidth of path $P_{1}$ is greater than that of $P_{2}$; for the purpose of speeding up the convergence rate of load balancing, we choose $P_{1}$ as routing path for newly triggered flowlet at a higher probability. In this section, we study the timeout setting problem when non-equal probability path selection is applied to flowlet switching based load balancing scheme.

Denote the probability that $P_{1}$ and $P_{2}$ are selected as the routing path for newly triggered flowlet as *p* and *q*, respectively. *p* and *q* meet the following conditions (to accelerate the convergence process of load balancing, *p* and *q* are usually set proportional to the bandwidth of the corresponding paths, i.e., $p=\frac{C_{1}}{C_{1}+C_{2}},q=\frac{C_{2}}{C_{1}+C_{2}}$), where

$$\left\{\begin{array}{c} p>0,\\ q>0,\\ p+q=1,\\ p\neq q. \end{array}\right.$$

It is easy to obtain the transition probability $P_{\left(n_{1},n_{2}\right)}^{1}$ as

$$P_{\left(n_{1},n_{2}\right)}^{1}=q\times \sum_{i\in F_{1}}\left(f\left(\lambda_{i}\right)+g\left(\lambda_{i}\right)\right),\;n_{1}\in \left[1,N\right].$$

Similarly, the transition probability $P_{\left(n_{1},n_{2}\right)}^{2}$ is

$$P_{\left(n_{1},n_{2}\right)}^{2}=p\times \sum_{i\in F_{2}}\left(f\left(\lambda_{i}\right)+g\left(\lambda_{i}\right)\right),\;n_{1}\in \left[0,N-1\right].$$

According to Equation (2), we can get the relationship of stationary probability between ideal state and that of any other state is

$$\begin{array}{cc} \frac{\pi_{\mu }}{\pi_{\mu +k}}=\frac{\pi_{\mu }}{\pi_{\mu +1}} & \times \frac{\pi_{\mu +1}}{\pi_{\mu +2}}\times ....\times \frac{\pi_{\mu +k-1}}{\pi_{\mu +k}}\\ ={\left(\frac{C_{1}}{C_{2}}\times \frac{q}{p}\right)}^{k} & e^{-\delta \sum_{j=1}^{k}\left(\frac{C_{1}}{(\mu +j)\gamma }-\frac{C_{2}}{(N-\mu -j+1)\gamma }\right)},\;k\in \left[1,N-\mu \right],\\ \frac{\pi_{\mu -k}}{\pi_{\mu }}=\frac{\pi_{\mu -1}}{\pi_{\mu }} & \times \frac{\pi_{\mu -2}}{\pi_{\mu -1}}\times ....\times \frac{\pi_{\mu -k}}{\pi_{\mu -k+1}}\\ ={\left(\frac{C_{1}}{C_{2}}\times \frac{q}{p}\right)}^{k} & e^{-\delta \sum_{j=1}^{k}\left(\frac{C_{1}}{(\mu -j+1)\gamma }-\frac{C_{2}}{(N-\mu +j)\gamma }\right)},\;k\in \left[1,\mu \right]. \end{array}$$

Following Equations (5) and (6), we have

(7) $$\delta_{\min}=\left\{\begin{array}{cc} 0, & \;\mathrm{if}\;qC_{1}=pC_{2},\\ \frac{\mu \left(N-\mu +1\right)\gamma \times \ln\left(\frac{C_{1}}{C_{2}}\times \frac{q}{p}\right)}{C_{1}\left(N-\mu +1\right)-C_{2}\mu }, & \;\mathrm{if}\;qC_{1}>pC_{2},\\ \frac{\left(N-\mu \right)\left(\mu +1\right)\gamma \times \ln\left(\frac{C_{1}}{C_{2}}\times \frac{q}{p}\right)}{C_{1}\left(N-\mu \right)-C_{2}\left(\mu +1\right)}, & \;\mathrm{if}\;qC_{1}<pC_{2}. \end{array}\right.$$

As long as δ > $\delta_{\min}$, adaptive load balancing can be achieved even with non-equal probability path selection. The derivation process is similar to Appendix A It can be seen that Theorem 1 is actually a special case with *p* = *q* = 0.5.

### 3.4. Timeout Setting in Multiple Parallel Load Balancing Paths

In this subsection, we study the problem of how to set δ to achieve adaptive load balancing when the load balancing parallel paths are multiple. When there are multiple parallel load balancing paths, each newly triggered flowlet may choose any of the paths as new routing path. As the number of multiple parallel paths increases, the Markov chain model encounters the state explosion problem and hence the computation of the transition probability matrix and the stationary distribution will become intractable. Alternatively, we leverage the results shown in Equation (7) to derive the threshold on δ.

Assume there are M parallel paths $P_{1}$, $P_{2}$, ..., $P_{M}$ with capacities $C_{1}$, $C_{2}$, ..., $C_{M}$, respectively. We divide all paths into two logical paths $P_{1}$ and $P_{2}$, where $P_{1}$ contains $P_{1}$ only and $P_{2}$ contains all the other paths. The bandwidth of $P_{1}$ and $P_{2}$ are $C_{1}$ = $C_{1}$ and $C_{2}$ = $\sum_{k=2}^{M}C_{k}$, respectively. With random path selection of the M paths, the probability that $P_{1}$ or $P_{2}$ is chosen as the routing path for newly triggered flowlet is *p* = $\frac{1}{M}$ and *q* = $\frac{M-1}{M}$, respectively. The number of flows mapping to $P_{1}$ can be calculated as μ = $\left\lfloor \frac{C_{1}}{C_{1}+C_{2}}\times N\right\rfloor$ when ideal load balancing effect is achieved. According to Equation (7), we can obtain $\delta_{\min}^{1}$ that specifies the lower bound on δ when ideal load balance effect is achieved between $P_{1}$ and $P_{2}$. After obtaining $\delta_{\min}^{1}$, we remove $P_{1}$ from the physical paths resulting in a new problem of how to achieve load balance between the remaining M−1 paths $P_{2},P_{3},\ldots ,P_{M}$. Similarly, we can obtain $\delta_{\min}^{2}$ by regarding $P_{2}$ as one logical path and all the other M−2 paths as another logical path. This procedure can be processed recursively until there are only two paths $P_{M-1}$ and $P_{M}$ left which yields $\delta_{\min}^{M-1}$. When ideal load balance effect is achieved between the M parallel paths, δ must be set as δ > $\delta_{\min}$, where

$$\delta_{\min}=\max\left\{\delta_{\min}^{k}\right\},\;k\in \left[1,M-1\right].$$

The detailed algorithm is described in Algorithm 2. The time complexity of the algorithm is 𝒪(M),
**Algorithm 2** An iterative algorithm for solving timeout setting problem with multiple parallel paths.**Input:**
One physical path set, $P\left(P_{1},P_{2},\ldots ,P_{i},\ldots P_{M}\right)(2\leq i\leq M$);One logic path set that contains physical path in P, $P_{k}\left(k=1\;or\;2\right)$;The bandwidth of physical path $P_{i}$, $C_{i}$;The bandwidth of $P_{k}$, $C_{k}\left(k=1\;or\;2\right)$;The total flow number, N;The number of flows mapping to $P_{1}$ when ideal load balancing effect is achieved, μ;The probabilities of $P_{1}$ and $P_{2}$ is chosen as the routing path for newly triggered flowlet, *p* and *q*;The *m-th* minimum value which must be less then δ, $\delta_{m}\left(1\leq m\leq M-1\right)$;
**Output:**   The maximum value of $\delta_{m}$, $\delta_{\min}$;
1:**for** m from 1 to M-1 **do**2:   *$P_{p}$ ← pick the first physical path in P;*3:   *$P_{1}$ ← ($P_{p}$) and $P_{2}$← P - $P_{1}$;*4:   *$C_{1}\leftarrow \sum_{P_{i}\in P_{1}}C_{i}$ and $C_{2}\leftarrow \sum_{P_{i}\in P_{2}}C_{i}$;*5:   *$\mu \leftarrow \left\lfloor \frac{C_{1}}{C_{1}+C_{2}}\times N\right\rfloor$;*6:   *$p\leftarrow \frac{1}{M+1-m}\;and\;q\leftarrow \frac{M-m}{M+1-m}$;*7:   *According to (7), we get the $\delta_{m}$;*8:   *N ← N-μ;*9:   *Remove $P_{p}$ from physical path set P;*10:**end for**11:*$\delta_{\min}$ ←* max*$\left(\delta_{m}\right)\;\left(1\leq m\leq M-1\right);$*12:**return** $\delta_{\min}$;

where M is the number of total parallel load balancing paths.

## 4. Performance Evaluation

In this section, we evaluate the performance of EasyLB both in symmetric topology and asymmetric topology with random path selection. Further, we evaluate EasyLB under non-equal path selection and multiple parallel paths in flowlet switching. Experimental results show that EasyLB achieves relatively ideal load balancing effect. Meanwhile, without maintaining explicit congestion information, EasyLB has the ability to handle asymmetry in the topology and achieves fast convergence of load balancing after link failures.

### 4.1. Asymmetric Topology with Random Path Selection

**Figure 5:**
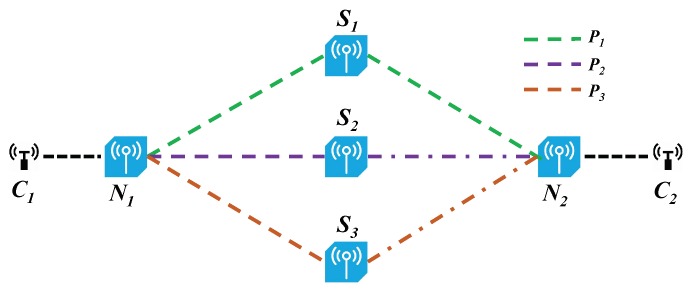
The testbed topology of wireless sensor networks.

**Figure 6:**
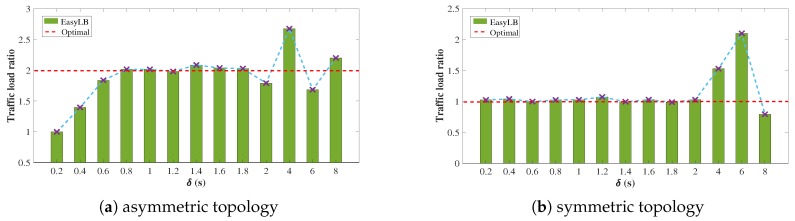
The convergent traffic load ratio of $P_{1}$ to $P_{2}$ under different timeout values.

The testbed topology of wireless sensor networks is shown in Figure 5. The testbed is based on SDN-WISE [33] which is a prototype system of SD-WSNs and uses OMNeT++ [34] simulator. We break down $P_{3}$ in the simulation of asymmetric topology and symmetric topology. The capacity of $P_{1}$ and $P_{2}$ are set to 20 and 10 Kbps, respectively. The delay difference between $P_{1}$ and $P_{2}$ is 0.01 s. The forwarding queues of the sensor nodes are running in a round-robin [35] fashion in our simulations to enhance the fairness of links. Ten long-lived TCP flows are generated that transmit from node $C_{1}$ to $C_{2}$. The randomly generated requests are distributed to $P_{1}$ and $P_{2}$. The average packet size of each flow is 80 Bytes. We run a basic flowlet switching process whose path selection is random, as described in Algorithm 1. We vary timeout value from 0.2 s to 8 s and get the convergent traffic load ratio of $P_{1}$ to $P_{2}$ as shown in Figure 6a. According to Equation (1), $\delta_{\min}$ equals to 0.64 s. When δ > $\delta_{\min}$, such as 1 s or 1.2 s, the system will achieve the ideal load balancing effect. However, if the value of timeout is set too large, such as bigger than 2 s, the granularity of flowlet switching based load balancing scheme is approximate to flow level and the traffic load ratio of $P_{1}$ to $P_{2}$ will be much more random. Similarly, if timeout is set too small, such as 0.2 s, it turns out to be packet-level load balancing and the traffic distributed onto this two paths is roughly the same.

**Figure 7:**
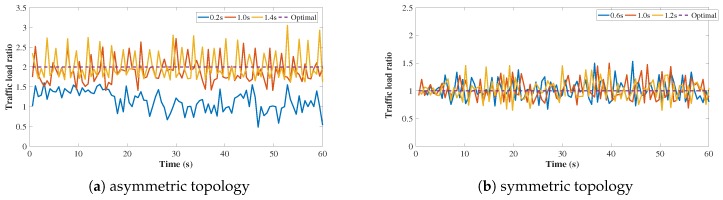
The traffic load ratio change of $P_{1}$ to $P_{2}$ under different timeout values.

In Figure 7, we set timeout to different values to show the real time traffic load ratio of $P_{1}$ to $P_{2}$. As shown in Figure 7a,b, the traffic load ratio fluctuates in both symmetric and asymmetric cases. As more flowlets are assigned to one path, congestion is more likely to occur on this path which results in the flowlet size reduction. Flows will transfer from a more congested path to a less congested one, and finally the size of flowlet on this two paths is balanced.

### 4.2. Symmetric Topology with Random Path Selection

We also evaluate the performance of EasyLB in symmetric topology, where the capacity of $P_{1}$ and $P_{2}$ are both set to 10 Kbps. The convergent traffic load ratio of $P_{1}$ to $P_{2}$ is shown in Figure 6b. According to Equation (1), $\delta_{\min}$ equals to 0 s. As depicted in Figure 6b, the traffic load ratio is approximately 1, which is the ideal load balancing effect in symmetric topology as long as the value of δ is not too large.

The load ratio change of $P_{1}$ to $P_{2}$ in symmetric topology is shown in Figure 7b. We can find that EasyLB converges to ideal load balance effect. We know that, when the number of flows in one path increases, this path becomes more congested and more packets are likely to be dropped. Packet loss in TCP brings about TCP timeout and a new flowlet will be generated, which forces flows switch to another path. When the system eventually reaches steady state, the congestion degree is almost the same.

### 4.3. Non-Equal Probability Path Selection

**Figure 8:**
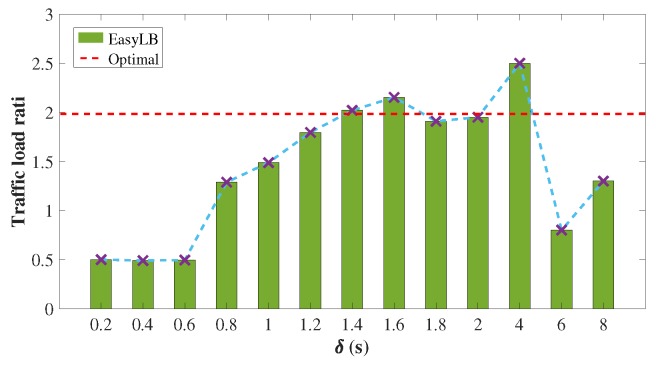
The convergent traffic load ratio of $P_{1}$ to $P_{2}$ with non-equal path selection under different timeout values.

To evaluate EasyLB with non-equal probability path selection, we set the path selection probabilities of $P_{1}$ and $P_{2}$ to $\frac{1}{3}$ and $\frac{2}{3}$, respectively. The bandwidth of $P_{1}$ and $P_{2}$ is set to 20 and 10 Kbps, respectively. According to Equation (7), $\delta_{\min}$ equals to 1.28 s. As shown in Figure 8, as long as δ is larger than $\delta_{\min}$, the traffic load ratio of $P_{1}$ to $P_{2}$ approximately approaches 2, which is the ideal load balancing effect. Note that, as δ is sufficiently large, it acts more like flow-level load balancing and hence the traffic load ratio of $P_{1}$ to $P_{2}$ has difficulty converging to 2, as shown in Figure 8. An interesting finding is that, although the path selection probabilities of $P_{1}$ and $P_{2}$ are inversely proportional to their bandwidth, EasyLB maintains its adaptive load balancing capability as long as the timeout is appropriately set.

### 4.4. Multiple Parallel Paths

**Figure 9:**
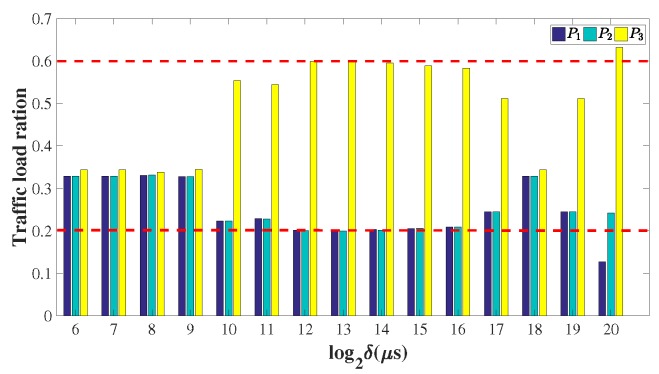
The convergent traffic load ratio of $P_{1}$, $P_{2}$ and $P_{3}$ under different timeout values.

In this subsection, we evaluate the performance of EasyLB in the presence of multiple paths. As shown in Figure 5, the client applications on sensor node $C_{1}$ randomly generate requests to server applications on $C_{2}$. The capacity of the three paths $P_{1}$, $P_{2}$, and $P_{3}$ are set to 6, 6 and 18 Kbps, respectively. We randomly assign new triggered flowlet to the three paths, thus the path selection probability of each path is $\frac{1}{3}$. According to Algorithm 2, $\delta_{\min}$ equals 0.88 s. As shown in Figure 9, when δ is set smaller than $\delta_{\min}$, EasyLB acts more like a packet-level load balancing scheme, therefore traffic loads of the three paths are almost the same. When δ is set larger than $\delta_{\min}$, EasyLB achieves the ideal load balancing effect, in which the traffic distributed to each path is proportional to its bandwidth. However, as noted above, when δ is too large, EasyLB evolves to flow level load balance scheme, thus the final traffic load ratio of the three paths is much more random.

### 4.5. React to Link Failure

**Figure 10:**
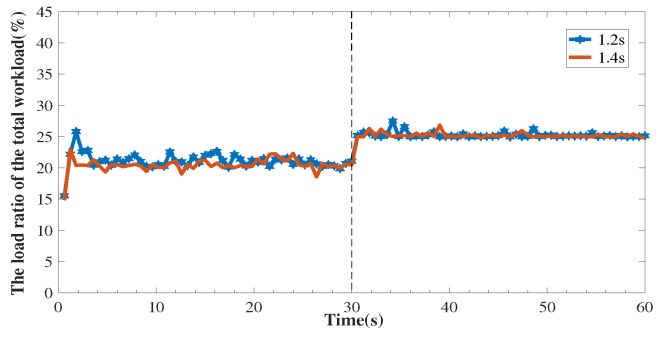
The traffic load change of $P_{2}$ under timeout value 1.2 s and 1.4 s. At t = 30s, a link down event occurs on $P_{1}$.

The capacity of three paths $P_{1}$, $P_{2}$, and $P_{3}$ are set to 6, 6 and 18 Kbps, respectively. We set δ to 1.2 s and 1.4 s. At first, the traffic is distributed over the three paths, and around 20% traffic is allocated to $P_{2}$. At t = 30 s, we manually break the link $\left(N_{1},S_{1}\right)$. As is shown in Figure 10, EasyLB reacts fast to this link failure event and around 25% of traffic is allocated to $P_{2}$ after the re-balance. This is because, once link failure happens in multi-paths case, the SDN controller can perceive it quickly and pushes the new multi-path decision into switches as a new group entry.

## 5. Conclusions and Future Work

We have studied the relationship between the timeout value in flowlet switching and the final state of load balancing with a stationary Markov chain. We further derived a theorem that specifies the sufficient condition on timeout to achieve ideal load balancing effect, which gives a comprehensive recommendation on setting timeout in different network environments. We also implemented flowlet switching based load balancing scheme called EasyLB in software defined networking by extending OpenFlow protocol. Experimental results show that, by setting the timeout following the theorem, EasyLB has adaptive load balancing ability both in symmetric topology and in asymmetric topology. The number of active flows was assumed to be static. However, in highly dynamic networks [36,37], such as vehicle-to-vehicle (V2V) networks, the number of flows changes rapidly. Moreover, the flow priority may affect the timeout value setting. How to deal with the dynamic arrival and departure of flows, as well as take the flow priority into account in timeout value setting, is left as future studies. 

## Appendix A The Derivation Process of Theorem 1

Taking the natural logarithm of both sides of Equations (5) and (6), we have

$$\begin{array}{cc} \ln\frac{\pi_{\mu }}{\pi_{\mu +k}} & =-\delta \sum_{j=1}^{k}\left(\frac{C_{1}}{(\mu +j)\gamma }-\frac{C_{2}}{(N-\mu -j+1)\gamma }\right) \end{array}$$

(A1) $$\begin{array}{c} +k\times \ln\frac{C_{1}}{C_{2}}>0,\;\;\forall \;k\in \left[1,N-\mu \right], \end{array}$$

(A2) $$\begin{array}{cc} \ln\frac{\pi_{\mu -k}}{\pi_{\mu }} & =-\delta \sum_{j=1}^{k}\left(\frac{C_{1}}{(\mu -j+1)\gamma }-\frac{C_{2}}{(N-\mu +j)\gamma }\right)\\ & +k\times \ln\frac{C_{1}}{C_{2}}<0,\;\;\forall \;k\in \left[1,\mu \right]. \end{array}$$

We can get $\frac{C_{1}}{(\mu +j)\gamma }-\frac{C_{2}}{(N-\mu -j+1)\gamma }$ is always less than zero for j∈[1,N−μ] and $\frac{C_{1}}{(\mu -j+1)\gamma }-\frac{C_{2}}{(N-\mu +j)\gamma }$ is always larger than zero for j∈[1,μ].

Thus, it can be derived from Equations (8) and (9) that δ should satisfy the following inequality as δ > $\delta_{\min}$ = $\max\{\delta_{\min1}=\max_{1\leq k\leq N-\mu }\left(f\left(k\right)\right),\delta_{\min2}=\max_{1\leq k\leq \mu }\left(g\left(k\right)\right)\}$ where

$$f\left(k\right)=\frac{k\times \ln\frac{C_{1}}{C_{2}}}{\sum_{j=1}^{k}\left(\frac{C_{1}}{(\mu +j)\gamma }-\frac{C_{2}}{(N-\mu -j+1)\gamma }\right)}$$

and

$$g\left(k\right)=\frac{k\times \ln\frac{C_{1}}{C_{2}}}{\sum_{j=1}^{k}\left(\frac{C_{1}}{(\mu -j+1)\gamma }-\frac{C_{2}}{(N-\mu +j)\gamma }\right)}.$$

If $C_{1}=C_{2}$, $\ln\frac{C_{1}}{C_{2}}=0$ and $\delta_{\min1}=\delta_{\min2}=0$, then $\delta_{\min}=0$. If $C_{1}>C_{2}$, $\ln\frac{C_{1}}{C_{2}}>0$ and $\delta_{\min1}<0,\;\delta_{\min2}>0$, then $\delta_{\min}$ = $\delta_{\min2}$. Similarly, if $C_{1}<C_{2}$, $\ln\frac{C_{1}}{C_{2}}<0$ and $\delta_{\min1}>0,\;\delta_{\min2}<0$, then $\delta_{\min}$ = $\delta_{\min1}$. Further, *f(k)* and *g(k)* are both decreasing, thus $\delta_{\min1}$ and $\delta_{\min2}$ get their maximum values when k=1. In conclusion, we can get the final results in Equation (1).
